# Assessment of nitrogen and phosphorus losses due to erosion in compost-treated and non-treated vineyard soils: effect of rainfall intensity

**DOI:** 10.1007/s10661-024-13269-8

**Published:** 2024-10-29

**Authors:** María Concepción Ramos

**Affiliations:** Department of Chemistry, Physics and Environmental and Soil Sciences, University of Lleida-Agrotencio CERCA Center, Lleida, Spain

**Keywords:** Compost amendment, Nutrient losses, Rainfall intensity, Rainfall erosivity, Soil loss, Soil water content

## Abstract

Vineyards in Mediterranean areas suffer from significant soil degradation through erosion, due to rainfall and soil characteristics, as well as soil management practices. Previous studies pointed out the nutrient losses produced by erosion and the benefits that some management practices could have on reducing erosion. This research tried to evaluate the effect of events of different intensities and to assess whether the beneficial effect of compost amendment may pose a potential risk of nutrient loss and environmental pollution in particular under high-intensity events. The study compared soil and nutrient losses in compost-treated and non-treated vineyard soils after rainfall events of different intensities analyzed over 2 years in two vineyards. Runoff samples were collected by triplicate in treated and non-treated soils. Sediment and nitrogen and phosphorous concentrations in the runoff samples were analyzed. The results reveal a reduction in runoff rates and an increase in soil water content in compost-treated soils, which represents a benefit for rainfed vineyards. Both nitrogen and phosphorus losses depended on rainfall characteristics. Although for low intensities there were no significant differences in the amount of nutrient lost by runoff in both treated and non-treated soils, nitrogen and phosphorus losses were higher after high-intensity rainfall events in compost-treated soils. With the expected increase in high-intensity rainfall events associated with climate change in the Mediterranean region, organic amendments should be applied in several splits or incorporated into the soil to avoid increased nutrient loss to water bodies.

## Introduction

Soil water erosion is one of the main soil degradation processes that causes a range of in-site and off-site damages and problems worldwide (Zuazo et al., 2011). High-intensity rainfall is often responsible for most soil losses although management practices can modulate the effects on cultivated lands (Dugan et al., 2022; Napoli et al., 2017). Vineyards are one of the typical land uses in the Mediterranean area and are mostly cultivated on bare soil due to water competition with weeds. The lack of soil cover was driven in the past by Spanish regulations, which did not allow irrigation until 1996. Nowadays, it is allowed in the event of unfavorable weather conditions (Ley 24/2003, BOE 165 de 11/7/2003), and it is regulated by each designation of origin (DO). In the Penedès DO, where the present study was carried out, irrigation is only permitted with the objective of improving grape quality when the hydric regime and the vine conditions do not allow reaching an optimal quality and in any case after veraison (Reglamento Denominación de Origen Penedès, BOE 167- 8/5/2009). However, irrigation is not always possible due to the scarcity of water to irrigate. Thus, rainfall is the only water supply, and although the total annual amount could cover the annual evapotranspiration demands, its distribution is very irregular, with the driest periods coinciding with those of highest water demand. In addition, storms occurring in summer and early autumn are usually of high intensity and a large amount of rainfall is lost as runoff. The conjunction of soil bare and high-intensity rainfall are the main factors that drive the high magnitude of erosion rates found in Mediterranean vineyards (De Santisteban et al., 2006; Raclot et al., 2009; García-Ruiz, 2010; Corti et al., 2011). Moreover, these erosion processes are being accelerated due to the leveling operations carried out to facilitate the mechanization of the labors. These operations alter the hydrological properties of the soil, which, in most cases, results in poorer soils with lower infiltration and soil water storage capacity (Misopolinos & Zalidis, 2002), leading to higher rates of runoff and erosion (Ramos & Martínez-Casasnovas, 2009). Soil erosion causes not only soil loss but also nutrient losses (mainly N, P, K, Ca, and Mg), being N and P as the main threats for the environment. In addition, in Mediterranean climates, most of annual nutrient losses are recorded in a few events (Ramos & Martínez-Casasnovas, 2009).

To improve soil conditions in the vineyards, farmers apply organic amendments such as compost, which act as organic fertilizers and increase the organic matter content. Among the different types of organic amendments (municipal soil waste compost, sewage sludge compost, farmyard manure, green manure, manure compost, and vermicompost), farmers in the area decided to use manure compost. These applications, made at the discretion of the farmer (in this case, farmers decided applications in alternated rows), can improve soil properties such as aggregate stability, porosity, infiltration, and water holding capacity of the soils (Weindorf et al., 2006; Fares et al., 2008; Morlat & Symoneaux, 2008; Rasoulzadeh & Yaghoubi, 2010; Brown & Cotton, 2011) and thus reduce runoff (Bean & Dukes, 2015). Nevertheless, long-term effects may be only observed under high application rates and applications may need to be repeated periodically to obtain observable effects (Aranyos et al., 2016), which leads to higher nutrient contents on the soil surface. However, not all elements incorporated into the soil with compost will be stored in the soils and/or mobilized in the same way. Nitrogen (N) tends to dissolve rapidly and can either leach towards groundwater or be transported in runoff, mostly in dissolved form (mainly as nitrate–N and ammonium-N, Wang et al., 2023b), and reach surface waters. Soil characteristics influence the N movement in addition to the water intake into the soil due to irrigation or to rainfall, but the amount N transported by runoff can represent a significant fraction. White et al. (2018) found nitrate loads in runoff generated after rainfall events that were one order of magnitude higher in cultivated than in non-cultivated areas, and higher nitrate loads in runoff than in groundwater. Phosphorus (P), on the contrary, tends primarily to bind to soil particles in the upper layers of the soil and to reach surface waters together with eroded soil particles, although it can also be carried away as dissolved form. In runoff from agricultural lands, P is mainly transported in particulate form (Sandström et al., 2020) and mainly linked to the smaller particles, which are more susceptible to be eroded (Osmond et al., 2019). It is hypothesized that the application of organic amendments, favoring some soil properties such as infiltration and water storage capacity, may reduce runoff but that the higher nutrient content on the soil surface may pose an increasing risk for the environment, in particular under high-intensity events, which are projected to increase under projected climate change scenarios. To further explore this aspect, this research aims to analyze the effects of compost application on N and P losses in compost-treated vineyard soils in a Mediterranean climate zone, the Anoia-Alt Penedès region (Northeast Spain), where compost has been observed to have a positive effect on soil water content (Ramos, 2017). Estimates on nutrient losses had already been made in the region considering a reduced number of rainfall events (Ramos & Martínez-Casasnovas, 2006), and this research attempts to complement the results to evaluate whether the positive effects that compost amendments may have on water retention may result in potential increases in nutrient losses as well as to know the role of rainfall characteristics and the antecedent soil water content on nutrient losses.

## Materials and methods

### Study area

**Fig. 1:**
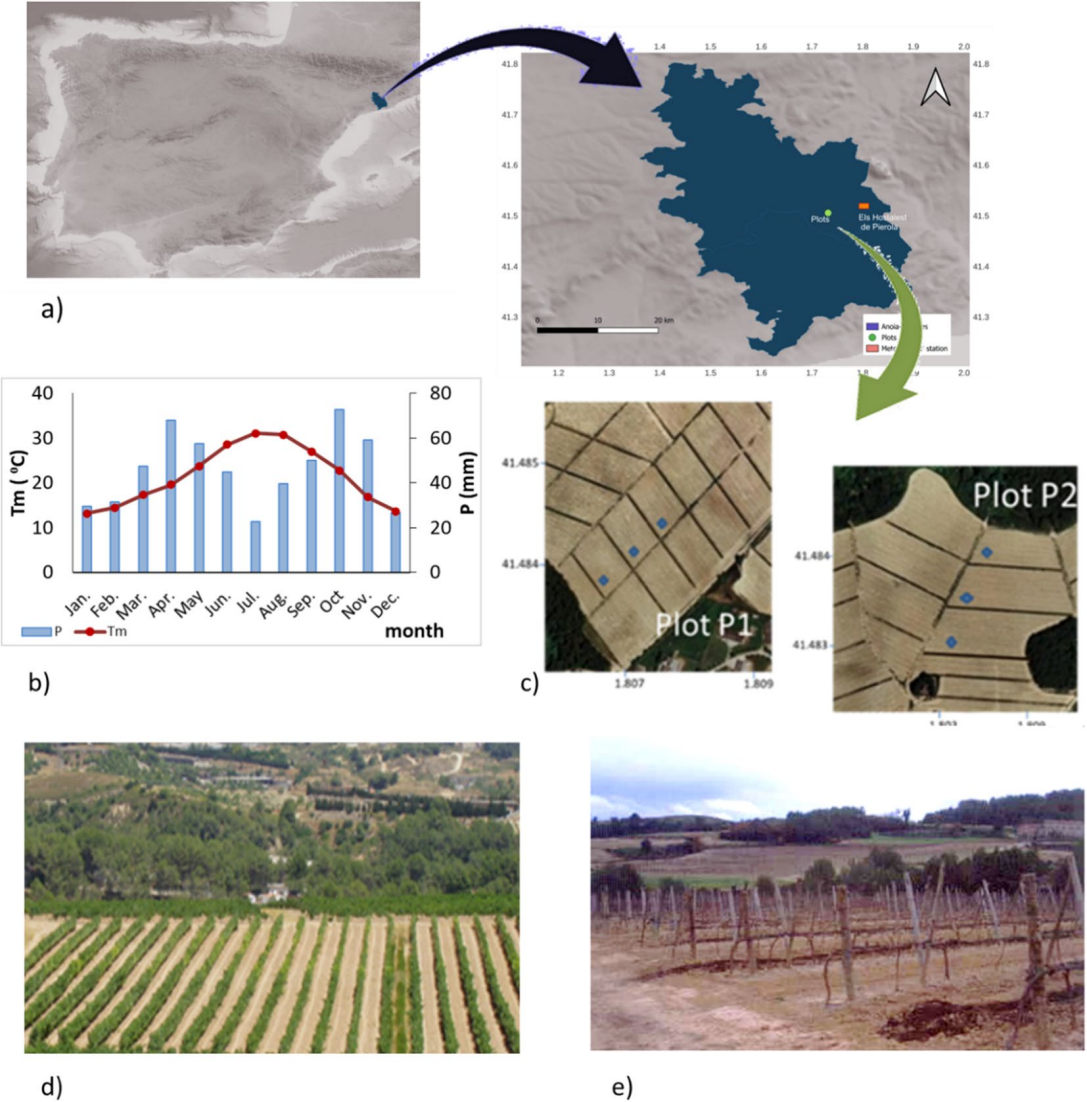
**a** Location of the study area (Anoia-Alt Penedès region, NE Spain) and **b** mean climate characteristics of the study area (mean monthly temperature (Tm) and monthly precipitation (P); **c** location of the sampling points in the plots; **d** view of plot 2 with a diversion terrace covered with vegetation; **e** view of plot 1 with compost amendment in alternated rows

The study was conducted in the Anoia-Alt Penedès region (NE Spain), about 35 km west of Barcelona (Fig. 1). The climate is Mediterranean with Maritime influence, with an average annual rainfall of about 540 mm (average of 43 years, 1960–2013, Ramos & Durán, 2014), mainly concentrated in two wet periods (spring and autumn) separated by hot and dry summers. The mean monthly temperature and precipitation in the study area, referred to the period 1998–2022, are shown in Fig. 1. High-intensity rainfall events are usually recorded in autumn (Ramos & Durán, 2014). The main land use in the area is vineyards, which occupy 80% of the cultivated land and cultivated under the DO Penedès. Most vineyards in the region are managed under rainfed conditions, and the soil is kept bare to reduce water competition with weeds, which are eliminated through tillage and/or herbicides.

### Soil characterization of the studied plots

The research was based on information collected from two vineyard plots (P1 and P2, Fig. 1). The vineyards were 1.18 and 1.58 ha, respectively. The average slope of the land is approximately 9%, quite uniform as the plots were leveled before plantation. The vines were planted 10 to 14 years before this research, in rows perpendicular to the maximum slope, 3.1 m between rows and 1.3 m between plants, with a trellis training system and with diversion terraces built perpendicularly to the main slope every 14 rows. The soils of the plots are classified as *Typic Xerorthents and Typic Calcixerepts* (SSS 2022), developed over marls, with moderately fine textures and few coarse elements on the surface, and with low organic matter content (< 20 gkg^−1^). Soil depth ranges from 0.80 to 1.15 m, with layers of marls causing significant restrictions on water infiltration.

Soil particle distribution (Gee & Bauder, 1986) was analyzed in each plot and is shown in Table 1. The soils have a relatively high silt content (particularly in plot P2), which favor surface sealing (Ramos et al., 2000), limiting water infiltration and increasing runoff generation.

**Table 1:** Soil particle size distribution (USDA) in the profile of each plot (mean ± standard deviation)

Soil depth	Sand (%)	Silt (%)	Clay (%)	Rock fragment (%)
Plot P1				
0–20 cm	48.5 ± 7.8	38.5 ± 9.2	13.0 ± 1.4	24.5 ± 4.1
20–50 cm	44.0 ± 2.8	42.0 ± 0.2	14.0 ± 2.8	37.4 ± 6.9
50–80 cm	47.5 ± 4.9	40.5 ± 3.5	12.0 ± 1.4	41.4 ± 12.3
Plot P2				
0–20 cm	31.0 ± 15.2	47.0 ± 13.5	14.5 ± 0.7	15.0 ± 1.4
20–40 cm	18.5 ± 3.5	61.0 ± 2.8	12.5 ± 2.1	11.0 ± 1.4
40–60 cm	19.5 ± 4.9	59.0 ± 19.5	13.0 ± 2.8	20.5 ± 6.4
60–115 cm	22.0 ± 8.0	57.0 ± 9.0	16.0 ± 2.2	18.0 ± 5.0

Compost from cow manure was applied to the fields at a rate of 40 Mg ha^−1^ in alternate rows and mixed in the top 25 cm of the soil with a chisel cultivator. The information included in this research refers to 2 years. In the first year of sampling, the soils had received a compost application one year prior to the analysis, and in the second year analyzed, the soils had received a second application 1 year prior to the analysis. The characteristics of the applied compost, which were taken from the information provided by the compost supplier (Organics S.A.), are shown in Table 2. In both plots, properties such as bulk density (Cresswell and Hamilton, 2002), water retention capacity at saturation, − 33 kPa and − 1500 kPa (Richard Plates), organic carbon (Alison, 1965), pH, and electrical conductivity (EC) as well as total N and total P in the soil surface layer were analyzed in soils with and without compost application. Soil pH was measured potentiometrically in H_2_O (soil to water ratio of 1:5), and EC was measured using KCl as a test solution (0.01 M, ratio 1:5). Nitrogen was determined following the Kjeldahl method (Sparks et al., 1996), and P was evaluated following the method proposed by Murphy and Riley (1962) after digestion with aqua regia. In addition, steady infiltration was evaluated using simulated rainfall in the field (as described in Ramos (2017)).

**Table 2:** Characteristics of the compost applied in the study (concentrations referred to dry weight)

Year	Organic matter (g kg^−1^)	Total-N (g kg^−1^)	Organic-N (g kg^−1^)	N-NH_4_ (g kg^−1^)	N-NO_3_ (g kg^−1^)	Total-P (g kg^−1^)	K (g kg^−1^)	pH_1:0.5_	EC_25_ (1:5) (dS m^−1^)
1	627	22.6	17.6	3.5	1.5	14.1	28.9	7.91	1.63
2	614	19.6	15.2	2.2	2.2	10.9	29.2	7.89	1.65

### Climatic information

Rainfall was recorded at 1-min interval using a tipping-bucket rain gauge station located in the study vineyard. The rest of climatic data were taken from the meteorological station of Els Hostalets de Pierola (lat: 41° 31′ 51.9″ N; long: 1° 48′ 29.3″ E; elev: 316 m a.s.l.), belonging to the automatic meteorological station network (XEMA) of the Institut Meteorologic de Catalunya (Spain) and located 6 km far from the study plots. For the recorded events, the mean and maximum intensity in 30 min was calculated, as well as their erosivity, estimated by the EI30 index (kinetic energy* maximum intensity in 30 min). The kinetic energy was estimated according to rainfall kinetic energy–intensity relationship proposed by Brown and Foster (1987).

### Runoff sample collection and analysis

Runoff samples were collected using Gerlach troughs in both treated and non-treated soils, which were installed at three locations in each plot. As diversion terraces divert runoff water, the collectors were placed in the three replications in the second and third row after the diversion terrace, in order to replicate the distance to the head of the plot. The collectors were 0.5-m wide, with covered tops to prevent direct rainfall entering the collectors. The Gerlach troughs were connected to 10 L deposits buried in the ground, in which runoff was collected. Runoff was collected after 23 rainfall episodes, recorded during the 2 years of the study. Runoff samples were collected after individual high-intensity rainfall events or/and after continuous rainfall recorded during several days that accumulated more than 15 mm. The deposits were large enough to collect all the runoff, and they were only completely filled after two events of high magnitude, in which that accumulated more than 100 mm (one period in each year). The total runoff collected was pulled from the collectors using a vacuum pump after homogenization. After measuring the volume, some aliquots were separated to determine the sediment and nutrient concentrations in the runoff. To determine the sediment concentration in runoff, the samples were dried at 105 °C and then weighed. Nitrogen was determined following the Kjeldahl method (Sparks et al., 1996), and total phosphorus was evaluated after acid digestion with ammonium persulfate and measured by colorimetry (Pierzynski, 2000). Samples were analyzed in duplicate, and each analysis included a blank and a standard sample as control. In addition to the runoff volumes collected in the Gerlach troughs, total runoff generated was estimated making a soil water balance taking into account the steady infiltration rate and soil water stored into the soil profile for each case (treated and non-treated soils) as well as crop evapotranspiration. Soil water was measured using TDR TRIME-FM (IMKO) probes, with measurements from 0 to 20 cm, 20 to 40 cm, 40 to 60 cm, and 60 to 80 cm at 15-day intervals in tubes installed in the field. The average soil water content in the profile was then calculated. The probe was calibrated in the lab and then checked in dry and wet periods with gravimetric soil moisture analysis.

Crop evapotranspiration was estimated by combining the potential evapotranspiration (ETo), estimated according to Penman Monteith model and the crop coefficients proposed by Allen and Pereira (2009). The Simpel Soil Water Model (https://www.hydrology.uni-kiel.de/de) was used to evaluate the water balance. The soil properties (soil water retention capacity at different potentials, root depth, maximum infiltration, and initial soil water content) of each plot and the time series for each year analyzed were adapted using information from Els Hostalest de Pierola meteorological station. Soil water storage was considered as control parameter and compared with the values measured in the field. For further analysis, the antecedent soil water content prior to the rainfall events analyzed was also estimated, as the mean value corresponding to the 10 days prior to the event. Runoff estimations were then used together with the sediment and nutrients collected in runoff to estimate total soil and nutrient losses. Mean nutrient concentration in runoff and nutrient losses in treated and non-treated soils were compared.

### Statistical analysis

The differences between plots and treatments (treated vs. non-treated soils) in soil and runoff were assessed with a test of means and analysis of variance, considering plot, treatment, and rainfall erosivity as factors. The response of soil to the repeated compost application was also evaluated (year factor). The effect of rainfall characteristics (total rainfall, rainfall intensity, and erosivity) on nutrient mobilization was evaluated in a correlation matrix in both treated and non-treated soils, using Spearman as in particular for runoff characteristics the data distribution was not normal. The antecedent soil water content prior to the events was also included in the analysis. The statistical analysis was carried out using Statgraphics Centurion.

## Results

### Changes in soil properties after compost application

The surface characteristics in treated and non-treated soils of the two plots analyzed are shown in Table 3. Compost application led to an increase in OM, a decrease in bulk density, and an increase in water holding capacity and steady infiltration rate, with a slightly higher improvement in plot P2 than in plot P1 (Table 3). The application of compost did not result in a significant change in pH, but there was an increase in the EC values and nutrient contents.

**Table 3:** Mean values and analysis of variance of soil surface characteristics of the studied plots as affected by plot (P1 and P2), treatment (compost treated (T) and non-treated (NT), and amendment year (Y1 and Y2). (P1-NT, plot P1 non-treated; P1-TY1, plot P1-treated, year 1; P1-TY2, plot P1–treated, year 2; P2-NT, plot P2 non-treated; P2-TY1, plot P2-treated, year 1; P2-TY2, plot P2–treated, year 2)

		OM (g kg^−1^)	N (g kg^−1^)	P (mg kg^−1^)	pH (ratio 1:5)	EC_25_ (ratio 1:5) (dS m^−1^)	Bulk density (kg m^−3^)	Sat. Hyd cond. (mm h^−1^)	Steady infilt. (mm h^−1^)	Water retention capacity (%)		
										Sat	− 33 kPa	− 1500 kPa
P1-NT		12.0	0.53	540	8.2	0.24	1610	16.5	23.1	40.0	33.0	12.1
P1-TY1		15.4	1.10	891	8.3	0.29	1580	22.5	26.6	41.6	34.9	12.2
P1-NTY2		12.5	0.62	528	8.3	0.30	1595	18.1	24.0	39.0	35.0	12.1
P1-TY2		17.0	1.10	1072	8.3	0.35	1460	24.0	30.2	41.9	36.0	12.6
P2-NT		4.8	0.40	478	8.1	0.24	1426	15.7	18.8	20.5	16.8	8.2
P2-TY1		12.0	1.03	673	8.6	0.29	1391	18.9	21.8	21.5	17.6	9.9
P2-NTY2		5.1	0.45	529	8.5	0.25	1436	16.7	17.9	21.0	17.5	8.5
P2-TY2		16.0	1.17	821	8.7	0.41	1380	20.3	24.8	21.7	20.4	10.2
Means												
Plot (P)	P1	14.1 b	0.97 b	963 b	8.3	0.33	1508 b	19.7	23.4 b	40.4 b	35.28b	12.4 b
	P2	9.5 a	0.76 a	739 a	8,6	0.39	1436 a	19.2	18.4 a	21.5 a	18.93a	9.2 a
Treatment (TR)	NT	8.6 a	0.45 a	523 a	8.4	0.25 a	1533 b	19.5	15.8 a	30.2	24.90 a	10.2
	T	15.0 b	1.27 b	851 b	8.4	0.36 b	1472 a	19.7	21.5 b	31.3	27.11 b	11.3
Year (Y)	Y1	13.7 a	1.13 a	766 a	8.4	0.31	1478	17.1	20.7	30.8	26.20 a	11.0
	Y2	16.3 b	1.40 b	936 b	8.5	0.41	1466	21.2	22.2	31.9	28.02 b	11.6
ANOVA factor												
P		***	**	***	***	ns	***	ns	**	***	***	***
TR		***	***	***	ns	**	*	ns	***	ns	***	ns
Y		***	**	***	ns	*	ns	ns ns	ns	ns	***	ns
P*TR		**	ns	*	ns	ns	*	ns	*	ns	ns	ns
P*Y		ns	ns	ns	ns	ns	***	ns	ns	ns	ns	ns

Significant differences ***: *p* < 0.01; **: *p* < 0.05: *: *p* < 0.1; *ns*, not significant. Number followed by different letters mean significant differences

### Characteristics of the recorded rainfall events

**Fig. 2:**
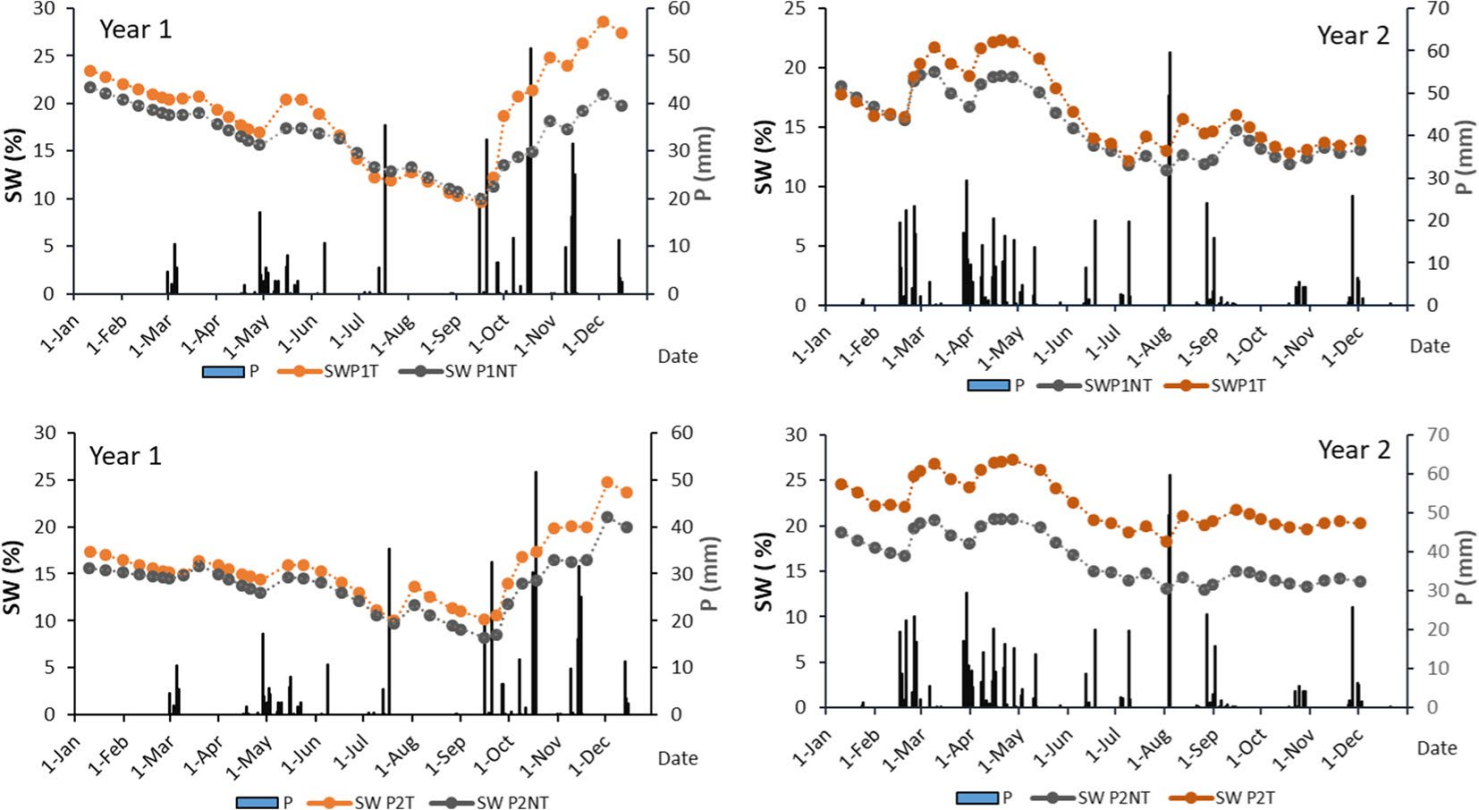
Precipitation recorded during the analyzed years and average soil water content in the profile in treated (T) and non-treated (NT) areas in plots P1 and P2

The 2 years included in the study differed in the total annual precipitation (435 and 587 mm, respectively, in year 1 and year 2) and also in its distribution throughout the year (Fig. 2). Nine erosive events were recorded in the first year, which accumulated between 17.2 and 104.8 mm, while in the second year, fourteen events were recorded, which accumulated between 19.2 and 108.6 mm (Table 4). Weak and heavy-intensity rainfalls were recorded in both years, with values that ranged from less than 2 to 41 mm h^−1^. Spring precipitations accumulated significant amounts of rainfall in events lasting more than one day but with relatively low rainfall intensity, while the highest rainfall intensities were recorded in autumn and summer events, respectively, in the first and the second year. Maximum intensities in 30 min of up to 60 mm h^−1^ were recorded in the first year and up to 84 mm h^−1^ in the second year, which implied rainfall with very high erosivity and annual erosivity. Due to the wide range of event erosivity, four classes were defined: class 1: events with rainfall erosivity < 30 MJ ha^−1^ mm h^−1^; class 2: events with rainfall erosivity between 30 and 100 MJ ha^−1^ mm h^−1^; class 3: events with rainfall erosivities between 100 and 200 MJ ha^−1^ mm h^−1^; and class 4, very high erosive events (> 200 MJ ha^−1^ mm h^−1^). Differences in soil and water content throughout the year were observed in both plots, in the treated and non-treated soils (Fig. 2). Soil water content was in general higher in treated soils, with soil water contents close to the values corresponding to the wilting point (values at − 1500 kPa, Table 3) in summer and over field capacity (values at − 33 kPa, Table 3) in winter, which led to differences in the antecedent soil water when the rainfall events took place (Table 4.) It can be observed that the changes in soil water content not always responded to the total amount of precipitation, in particular after the high-intensity events in which a high proportion of water could not infiltrate.

**Table 4:** Rainfall characteristics of the analyzed events and average antecedent soil water content in compost-treated (T) and non-treated (NT) soils of plots P1 and P2. (Prec, rainfall; Im, mean rainfall intensity; Imax30, maximum rainfall intensity in 30-min period; EI30, rainfall kinetic energy* maximum rainfall intensity in 30-min period)

Event		Rainfall characteristics			Antecedent soil water content			
	Prec (mm)	Im (mm h^−1^)	Imax30 (mm h^−1^)	EI30 (MJ ha^−1^ mm h^−1^)	P1-T (%)	P1-NT (%)	P2-T (%)	P2-NT (%)
Year 1								
8/3	17.8	2.2	7.2	24.5	20.42	18.76	14.98	14.84
8/5	17.2	4.2	8.7	16.9	16.94	15.62	14.33	12.90
24/5	37.6	4.9	9.6	8.3	20.43	17.45	15.90	15.50
19/7	41.8	16	24.5	83.5	12.28	11.89	11.16	10.62
17/9	54.2	3.3	36.4	139.3	9.62	9.50	10.20	8.19
9/10	37.6	16.2	34.2	63.3	20.75	14.25	15.57	11.84
20/10	104.8	19.8	60.7	928.9	21.48	14.96	17.34	14.30
10/11	83.2	12.0	25.4	140.9	23.95	17.27	20.07	16.28
17/12	17.4	1.05	8.7	9.5	27.41	19.77	23.67	19.92
Year 2								
22-Feb	53.0	1.67	13.3	28.6	15.83	15.63	19.98	16.49
27-Feb	44.2	3.4	7.9	66.9	19.22	18.85	23.61	19.72
30-Mar	76.8	4.0	6.0	95.5	19.31	16.71	22,37	17.96
08-Apr	20.8	2.5	8.4	13.4	21.62	18.59	24.58	20.17
16-Apr	53.6	3.3	22.0	96.6	22.14	19.19	25.30	20.89
15-May	67.8	1.6	8.4	98.4	20.73	17.94	24.32	19.81
20-June	30.6	4.8	50.8	166.0	14.07	13.43	18.70	15.04
15-July	27.0	3.1	24.9	44.9	12.14	11.83	17.12	13.57
4-Aug	108.6	12.9	84.2	1535.2	12.99	11.33	15.66	13.04
29-Aug	25.4	41.0	48.0	955.3	14.51	11.92	14.94	12.92
3-Sept	21.7	5.7	15.6	65.2	14.67	12.27	15.01	12.99
16-Sept	34.4	16.1	18.6	114.2	16.06	14.69	16.49	14.55
2-Nov	19.2	0.99	5.7	8.8	13.73	12.83	15.08	13.01
5-Dec	42.2	1.5	13.2	94.7	15.07	13.93	16.07	14.13

### Runoff generation and transported of soil and nutrients by runoff

Table 5 shows the characteristics of the runoff generated in treated and non-treated soils in both plots after the 23 events that generated runoff. Runoff ratios (runoff/rainfall*100) varied between events, ranging between less than 1% and more than 60%. There were no significant differences between plots and although in general, runoff rates were lower in treated than in non-treated soils (17.5 vs. 19.1% and 20.45 vs. 25.1%, respectively, for plot 1 and 2), which was consistent with the increase in soil water content mentioned above. The differences were not significant. Nevertheless, significant differences appeared between events with different erosivity.

**Table 5:** Mean runoff ratios and sediment concentrations in runoff recorded in plots P1 and P2 in compost-treated (T) and non-treated (NT) soils in the analyzed rainfall events

Events	Accmul. Prec (mm)	Runoff ratio				Sed. Conc			
		P1-T (%)	P1-NT (%)	P2-T (%)	P2-NT (%)	P1-T (g L^−1^)	P1-NT (g L^−1^)	P2-T (g L^−1^)	P2-NT (g L^−1^)
Year 1									
8-Mar	17.9	8.78	9.19	9.95	14.93	2.2	0.8	1.15	0.8
8-May	17.2	5.81	7.06	9.09	10.91	1.8	1.7	2.9	1.9
24-May	37.6	1.57	1.7	1.18	3.14	3.8	1.2	3.9	2.0
19-Jul	41.8	5.84	7.73	9.92	19.58	3.0	2.2	1.9	2.9
17-Sep	54.2	21.03	27.16	27.00	30.00	15.0	16.5	10.7	13.8
9-Oct	37.6	13.44	14.21	21.15	26.43	2.5	4.1	6.0	14.6
20-Oct	104.8	56.14	57.8	51.48	57.01	3.7	6.3	8.9	11.9
10-Nov	83.2	18.10	19.30	19.00	21.80	7.8	9.1	8.9	10.4
17-Dec	17.4	8.50	8.43	8.40	9.10	1.6	2.5	3.9	4.2
Year 2									
22-Feb	53.0	1.57	1.70	1.18	3.14	1.3	4.8	3.0	6.0
27-Feb	44.2	0.83	1.65	6.21	7.45	1.2	3.2	2.0	4.01
30-Mar	76.8	8.78	9.19	9.95	14.93	0.4	0.4	0.7	1.6
08-Apr	20.8	13.44	14.21	21.15	26.43	0.4	0.5	0.3	2.3
16-Apr	53.6	5.84	7.73	9.92	19.58	0.5	0.6	0.2	1.6
15-May	67.8	35.87	36.59	36.59	39.63	0.6	1.1	0.3	1.3
20-Jun	30.6	35.29	40.00	42.34	50.00	6.3	10.4	6.1	19.0
15-Jul	27.0	5.81	7.06	36.36	40.91	7.7	5.8	9.0	15.3
4-Aug	108.6	58.94	60.59	64.46	69.89	20.3	16.1	6.0	12.3
29-Aug	25.4	21.03	27.16	27.00	30.00	2.5	4.1	6.0	14.6
03-Sep	21.7	37.54	41.31	32.96	37.98	4.1	2.5	7.1	14.0
16-Sep	34.4	7.72	8.13	2.03	8.13	1.2	4.6	2.7	3.7
2-Nov	19.2	8.10	9.30	9.00	11.80	1.2	1.1	1.8	1.5
5-Dec	42.2	21.49	22.51	14.22	23.70	1.5	2.3	1.9	1.8

**Fig. 3:**
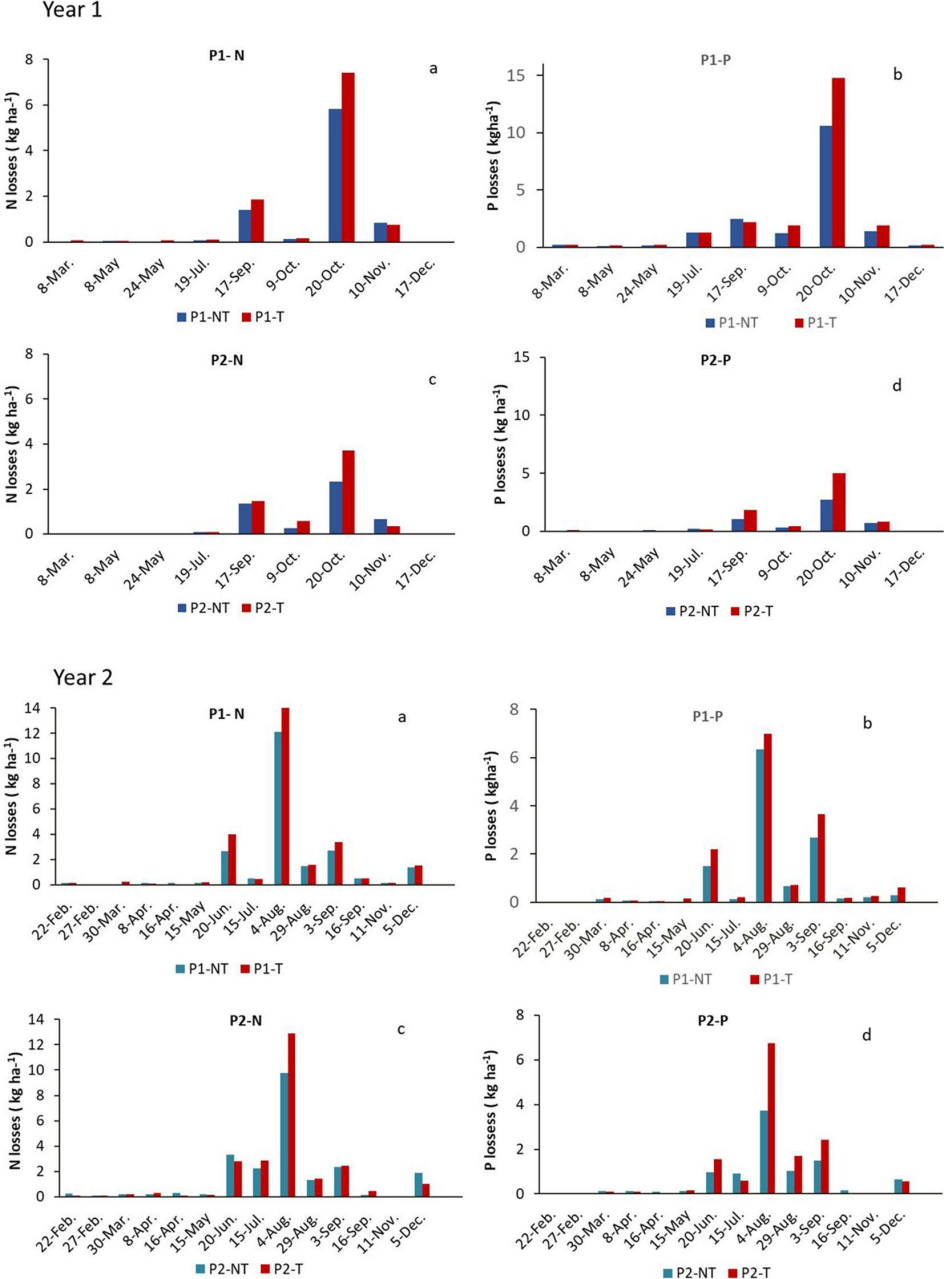
Total N (**a, c**) and total P (**b, d**) losses in plots P1 and P2 after the recorded events in compost-treated (T) and non-treated (NT) soils. (*means significant difference in nutrient losses between treated and non-treated soils)

Regarding sediment concentrations in runoff, the highest values were recorded in the autumn events of year 1 and in the late spring and summer events of year 2 (Table 5). Contrary to what was observed for runoff ratios, sediment concentration in runoff was in most cases higher in non-treated than in treated soils. There were some differences between plots, with higher soil concentrations in runoff almost always recorded in plot P2 than in plot P1 (significant differences at 90%) and higher in non-treated than in treated soils. The differences between treated and non-treated soils were higher for the intermediate erosive events (erosivity classes 2 and 3). As for nutrient concentrations in runoff, higher concentrations of N and P were observed in treated than in non-treated soils (significant at 90%) and with higher levels in plot P1 than in plot P2 (Table 6), in agreement with the higher N and P contents reached in the soils, although the differences were not significant. Nitrogen concentration ranged from less than 1 to more than 55 mg L^−1^, with significantly higher values (at 95%) in treated than in non-treated soils after the high erosive events (events in erosivity classes 3 and 4). Phosphorus concentration ranged from 0.5 to 39.1 mg L^−1^ in non-treated soils and between 1.2 and 52.6 mg L^−1^ in treated soils, with significant differences between plots and treatments, and with significantly higher values in treated soils under the high erosive events (erosivity classes 3 and 4). Figure 3 shows the total N and P losses, estimated by taking into account runoff rates and the concentration of sediments and nutrients in the runoff. It can be observed that a small number of events recorded high N and P losses, which determined the annual loss of nutrients in both treated and non-treated soils. Significantly higher N and P losses were recorded in treated compared to non-treated soils in the most erosive events (events included in erosivity class 4). The results of the analysis of variance for runoff, sediments, and nutrient concentrations in runoff and nutrient losses are summarized in Table 7.

**Table 6:** Mean nutrient (N and P) concentrations in runoff after the analyzed rainfall events recorded in in plots P1 and P2 in compost-treated (T) and non-treated (NT) soils

Events	Plot P1				Plot P2			
	N conc P1-T (mg L^−1^)	N conc P1-NT (mg L^−1^)	P conc P1-T (mg L^−1^)	P conc P1-NT (mg L^−1^)	N conc P2-T (mg L^−1^)	N conc P2-NT (mg L^−1^)	P conc P2-T (mg L^−1^)	P conc P2-NT (mg L^−1^)
Year 1								
8-Mar	5.3	1.7	13.1	12.0	0.3	0.9	5.3	0.4
8-May	4.9	3.4	13.2	6.6	1.9	1.2	4.9	3.9
24-May	12.5	3.1	35.5	22.3	2.6	2.7	12.5	9.2
19-Jul	4.2	2.2	52.6	39.1	2.0	1.1	4.2	2.7
17-Sep	12.5	12.6	19.1	16.9	10.0	8.3	12.5	6.4
9-Oct	3.3	2.2	37.7	23.1	7.4	2.7	5.9	3.3
20-Oct	12.6	9.6	25.1	17.5	6.9	3.9	9.3	4.6
10-Nov	11.2	10.9	12.5	8.6	4.7	6.8	11.2	7.2
17-Dec	1.3	0.8	14.2	10.9	2.2	2.1	2.1*	0.8
Year 2								
22-Feb	16.8	13.3	2.7	2.3	18.2	16.8	2.7	2.3
27-Feb	6.8	4.2	1.5	1.1	4.2	4.0	1.5	0.6
30-Mar	3.2	0.8	2.8	1.9	2.4	1.6	1.2	1.0
08-Apr	1.6	1.8	1.2	1.0	3.2	1.6	0.9	1.1
16-Apr	1.5	3.2	1.6	1.1	2.6	3.1	0.5	1.1
15-May	3.7	2.3	2.5	0.5	3.2	2.9	2.4	2.1
20-Jun	56.7	33.1	31.0	18.8	33.0	33.2	18.2	9.8
15-Jul	30.0	24.9	12.7	7.4	35.9	24.9	7.4	10.3
4-Aug	26.0	18.4	10.9	9.7	18.4	10.2	9.7	4.9
29-Aug	29.8	21.7	13.8	9.7	21.0	18.0	25.0	13.7
03-Sep	22.0	16.0	18.1	16.0	17.4	16.0	18.1	9.8
16-Sep	26.7	24.3	10.0	8.5	47.3	24.3	10.0	7.2
2-Nov	5.7	4.0	10.9*	7.0	0.8	0.7	1.7	0.5
5-Dec	16.8	14.7	6.6*	3.2	18.7	16.8	9.6	6.6

**Table 7:** Analysis of variance of runoff rates, sediment concentration in runoff, nitrogen and phosphorus concentration in runoff, and nitrogen and phosphorus losses as affected by plot, treatment (compost treated (T) and non-treated (NT)), and rainfall erosivity (erosivity classes 1–4) and the interactions between factors

	Runoff rates	Sediment conc	N conc	P conc	N losses	P losses
	(%)	(g L^−1^)	(mg L^−1^)	(mg L^−1^)	(kg ha^−1^)	(kg ha^−1^)
Means						
Plot (P)						
P1	22.77	5.14 a	13.81	13.16 b	2.46	2.23 a
P2	26.71	6.80 b	11.38	7.26 a	1.82	1.25 b
Treatment (TR)						
NT	26.40	6.67 b	10.75 a	8.16 a	1.98a	1.46 a
T	23.08	5.28 a	14.44 b	12.25 b	2.30 b	2.02 b
Erosivity class (E)						
1	8.53 a	2.16 a	4.69 a	7.19	0.08 a	0.10 a
2	19.03 b	3.82 a	8.18 b	9.12	0.64 ab	0.63 ab
3	22.95 b	7.69 b	21.13 c	11.70	1.62 b	1.14 b
4	48.46 c	10.23 c	16.38 c	12.83	6.22 c	5.09 c
ANOVA factors						
P	ns	*	ns	***	s	***
T	ns	*	*	**	*	*
E	***	***	***	ns	***	***
P*TR	ns	*	**	**	ns	ns
P*E	ns	*	ns	ns	ns	*
T*E	*	**	**	**	*	*

Significant differences; ***: *p* < 0.01; **: *p* < 0.05: *: *p* < 0.1; *ns*, non-significant; values followed by different letters are significantly different

### Relationship between rainfall characteristics and soil and nutrient losses

Table 8 shows the correlation matrix between sediment and nutrient concentrations in runoff with rainfall characteristics. In both treated and non-treated soils, a significant correlation was observed between total soil and nutrient losses recorded in each event and total rainfall, intensity, and rainfall erosivity, while the correlation was only significant for total rainfall and maximum intensity when soil and nutrient concentrations in runoff were considered. Regarding the effect of the antecedent soil water content, it was observed that total nutrient and soil losses were not significantly correlated with the antecedent soil water content, in both treated and non-treated soils. However, sediment and nutrient concentrations in runoff were significantly negatively correlated with the soil water content in non-treated soils.

**Table 8:** Correlation coefficients between sediment and nutrient concentrations in runoff and total soil and nutrient losses with rainfall characteristics and with runoff and antecedent soil water content generated in treated (T) and non-treated (NT) soils. (Concentrations in runoff: N (NC)), P (PC), and sediment (SC); total losses: total N (NL), total P (PL), and soil (SL); event precipitation (Prec), maximum intensity in 30 min (Imax30), rainfall erosivity (EI30), and antecedent soil water content (SW))

	NL-T (kg ha^−1^)	PL-T (kg ha^−1^)	SL-T (Mg ha^−1^)	NC-T (mg L^−1^)	PC-T (mg L^−1^)	SC-T (g L^−1^)
Prec (mm)	0.5546	0.6131	0.5699	− 0.3124	− 0.2504	− 0.1908
	***	***	***	*ns*	*ns*	*ns*
EI30 (MJ ha^−1^ mm h^−1^)	0.6057	0.6685	0.6215	0.1132	0.1952	0.0567
	***	***	***	*ns*	*ns*	*ns*
Imax30 (mm h^−1^)	0.5675	0.6094	0.5950	0.6555	0.7153	0.5505
	***	***	***	***	***	***
Runoff-T (mm)	0.8950	0.9412	0.8977	0.0262	0.1762	0.1451
	***	***	***	*ns*	*ns*	*ns*
SW-T (%)	− 0.2382	− 0.2410	− 0.2254	− 0.3207	− 0.2291	− 0.3357
	*ns*	*ns*	*ns*	*ns*	*ns*	*
	NL-NT (k gha^−1^)	PL-NT (kg ha^−1^)	SL-NT (Mg ha^−1^)	NC-NT (mg L^−1^)	PC-NT (mg L^−1^)	SC-NT (g L^−1^)
Prec (mm)	0.4361	0.5478	0.6135	− 0.2889	− 0.2232	− 0.0720
	**	***	***	*ns*	*ns*	*ns*
EI30 (MJ ha^−1^ mm h^−1^)	0.5148	0.6138	0.6664	0.1230	0.2477	0.2834
	***	***	***	*ns*	*ns*	*ns*
Imax30 (mm h^−1^)	0.5360	0.5556	0.5571	0.6659	0.6792	0.6834
	***	***	***	***	***	***
Runoff-NT (mm)	− 0.3608	− 0.3446	− 0.3204	− 0.5531	− 0.5380	− 0.5443
	*	*	*ns*	***	***	***
SW-NT (%)	− 0.3608	− 0.3446	− 0.3204	− 0.5531	− 0.5380	− 0.5443
	*	*	*ns*	***	***	***

Correlation significance: ***: *p* < 0.01; **: *p* < 0.05: *: *p* < 0.1; *ns*, not significant

## Discussion

### Changes in soil properties due to amendment

The results show the effects of compost amendment on soil properties, mainly in soil organic matter content (OM), EC, and nutrients (N and P), in particular in the second year after the second compost application (Table 3 and Table 7). The increase in OM was more notable in plot P2 than in plot P1, being plot P2 the one with the lowest OM content before the amendment. The increase in OM and N agrees with that found in other experiments in which compost was used as fertilizer (Duddigan et al., 2021; Reimer et al., 2023). The increase in P on the soil surface was even more evident in both plots after the successive applications, which was consistent with the main process for which P is fixed on the soil surface (Ahmed et al., 2023) and with results found in other research (Maltais-Landry et al., 2015). The increase in EC, due to compost addition, should be a factor to consider when applications are repeated since high salinity levels could affect vine development (Carter, 1982). This effect is not evaluated in this research, and it may be a topic to explore in future research.

After amendment, a greater amount of water could infiltrate even when higher rainfall intensity was recorded, as a result of the increase in steady infiltration rate (Table 3). In addition, compost amendments could have favored the proportion of macro aggregates associated to the increase in OM (Yu et al., 2012). Higher infiltration meant lower runoff rates and, in addition, more water was accumulated in the soil profile (Fig. 2), with a consequent increase in water availability for the crop in treated soils compared to non-treated soils. This increase in soil moisture was indicated by Burg et al. (2018) as one of the positive effects of using compost as some of the mulching materials to protect soils and reduce soil erosion, and Ramos (2017) confirmed the effect on the water available in the soil for the crop, which in addition will have an effect on grape yield and quality (Badalíková et al., 2022).

Regarding nutrient content in the soils, it was observed that N and P concentrations increased significantly after compost application (Table 3). Nitrogen in treated soils was more than double after compost application, with higher values in plot P1 than in plot P2, which continued increasing after the second application. For phosphorus, there was also an increase, reaching values near 2 and 1.6 times higher in plot P1 and plot P2, respectively, after the second application. This means on the one hand, the need to apply lower rates of additional fertilization but on the other hand, higher N and P content on the surface could be released and transported by runoff, both dissolved and (especially for P) bound to the detached soil particles.

### Runoff and soil and nutrient losses in compost treated and non-treated soils after rainfall events

The 2 years included in the study are representative of the rainfall recorded in the area (Ramos & Durán, 2014). The total annual precipitation and its distribution throughout the year differed between both years but low and high intensity rainfall events were recorded in each of them. Annual rainfall erosivity in the first year (EI30 index = 1415 MJ ha^−1^ mm h^−1^) was close to the annual average in the area (Ramos & Durán, 2014) and mainly due to one event recorded in autumn (928.9 MJ ha^−1^ mm h^−1^, which represented 65.6% of the annual erosivity). In the second year, rainfall erosivity was more the double, due to a couple of extreme summer rainfall events (with 1535 and 955 MJ ha^−1^ mm h^−1^, respectively, which represented 73.6% of annual erosivity). In areas with Mediterranean climate, it is quite common that one or two events account for a high percentage of the annual erosivity (Ramos & Martínez-Casasnovas, 2009). Nevertheless, other high EI30 events were recorded in the period analyzed (two events in the first year and four in the second year, with values close to or higher than 100 MJ ha^−1^ mm h^−1^). In some cases, despite recording small rainfall amounts, they had high intensities in short time periods, which resulted in moderate to high erosivity values (Table 4). Thus, the period analyzed could provide information on the effect of high intensity on soil and nutrient losses in runoff.

The highest runoff rates were recorded in the highest magnitude events recorded in autumn in year 1 and in summer of year 2 (close to 60 and 70% of rainfall was lost as runoff, respectively, in these events), but there were also high runoff rates (> 25%) in events that accumulated less rainfall, as was the case of some events recorded in September and October in year 1 and in May and June in year 2. On these dates, the soil was not saturated as denoted by the existing soil moisture (Table 4), which was lower than the soil water content at saturation (Table 3) on both plots in year 1 and on the untreated plot in year 2. Thus, in those cases, runoff probably occurred mainly due to the high intensity of the rainfall, which was higher than infiltration rate. Runoff rates were, on average, higher in non-treated than in treated soils, and the differences were higher in plot P2 than in plot P1 and depended on the event. Differences in runoff rates higher than 50% between treated and non-treated soil were observed, although these differences occurred in cases where the runoff coefficients were very low, in such instances, small variations can lead to significant percentage differences, but these do not necessarily correspond to substantial runoff quantities. However, in the events that recorded the highest runoff rates, the differences ranged between 10 and 20%. The differences in runoff rates between plots were in accordance with the lower steady infiltration in plot P2, justified by the lower coarse element and OM content and the higher silt content, which make the soil highly susceptible to seal (Ramos et al., 2000), limiting infiltration and favoring runoff.

The highest sediment concentrations in runoff were recorded in the autumn events of year 1 and in the late spring and summer events of year 2, which were those with the highest rainfall intensity. Sediment concentrations in runoff reached in those events up to 15 and 20 g L^−1^, while in some spring events, sediment concentrations were lower than 1 g L^−1^. It can be observed that in some cases, sediment concentration in runoff was higher in plot 1 than in plot 2. The result could be due to a dilution effect as runoff rates were higher in plot P2 in plot P1, in addition to the fact that high rainfall intensity rainfalls could favor the aggregate breakdown. Although a large amount of soil may be released in the early stages of the erosion process, the higher susceptibility to sealing may subsequently lead to a decrease in soil detachment. In Mediterranean areas, the combination of high rainfall intensity rainfalls, high erodible, and bare soils have been pointed out as responsible for significant erosion rates (Garnier et al., 2024), and the effect of rainfall intensity on runoff and on soil losses is in agreement with the results observed by Biddoccu et al. (2014) in vineyards.

As for nutrient concentrations in runoff, higher concentrations of N and P were observed in treated than in non-treated soils and with higher levels in plot P1 than in plot P2 (Table 6) in agreement with the higher N and P contents reached in the soils. The highest concentrations for both elements simultaneously were found in the events recorded on September 17, in year 1, and on June 20 and July 15, in year 2, although similar or higher N concentration was found on other dates in year 1 (e.g., May 24, October 9, and October 20) and also higher P concentrations in other dates (July 19, October 9, and October 20). Although N tends to dissolve rapidly and will be mainly transported as dissolved, the fact that it is applied in organic form means that it can be also transported by runoff as organic nitrogen. Although under different conditions but is soil rich in organic matter, Taylor et al. (2015) found that dissolved organic nitrogen in stream water represented a high percentage of the dissolved N exported and that N-nitrate export was very low. However, P may be susceptible to being carried away by runoff dissolved and bound to soil particles (Sharpley et al., 2008). After compost application, P concentrations in runoff were higher in treated than in non-treated soils (Table 6) in almost all events. The highest N and P concentrations were not recorded in the most erosive events, which could be justified by a dilution effect, since the most erosive were the ones that also accumulated the highest amount of water.

Regarding the effect of the antecedent soil water content, it was observed that sediment and nutrient concentrations in runoff were significantly negatively correlated with the soil water content in non-treated soils. This means that under non-treated soils, nutrient detachment and nutrient delivery appear to be greater under drier conditions. The effect of the antecedent soil water content has been discussed by Truman et al. (2011), who reported works with opposite effects. More recently, Wang et al. (2023a) indicated that antecedent soil water content influences splash, with splash erosion rates being higher when the earlier stages occurred in dry than in wet soils. In the case of the study, the results would be in line with those that reported that increasing soil water content significantly decreases soil detachment, runoff, and sediment delivery. In both treated and non-treated soils, the sign of the correlation was the same, although it was only significant in the non-treated soils. That difference could be due to the difference in soil water content. However, the result could be an artifact effect because the events of higher intensity and erosivity were recorded in summer (in mid-September and August, respectively, in both years analyzed) when soil water content was lower.

Most of the nutrient losses were due to 50% of the events recorded (Fig. 3), and in particular due to those recorded in the events of greatest erosivity. This agrees with results found previously by other authors who indicated that few annual intense storm events are usually responsible for the majority of the phosphorus (P) that reaches water bodies (Jordan et al., 2007; Sharpley et al., 2001). In this respect, Sharpley et al. (2008) indicate that P release increases with storm size and that few major storm events can generate most of total P losses in agricultural watersheds. In addition, the effect of rainfall intensity on nutrient losses has been indicated by different authors under different crops in arable lands (An et al., 2023; Napoli et al., 2017; Yang et al., 2023) and under other land uses (Ramos et al., 2019; Wang et al., 2019). Napoli et al. (2017) confirmed significant correlations between total soil and nutrient losses with rainfall amount and rainfall erosivity. Importantly, in the case study, the highest erosivity (EI_30_ = 928.9 and 1535.2 MJ ha^−1^ mm h^−1^) was also recorded in the events that accumulated the highest amount of water (104.8 and 108.6 mm), which drove most of N and P losses in each year of the study period. In other high erosive events, such as the one recorded on August 29 in year 2 (EI_30_ = 955.3 MJ ha^−1^ mm h^−1^), which accumulated less water, a high concentration of N and P was observed (Table 5), although the total loss of N and P was lower due to the lower amount of runoff produced. In addition, after the high-intensity rainfall events, total N and P losses were higher in treated than in non-treated soils, despite the reduction in runoff rates. The greater nutrient accumulation on the surface after the amendment may account for the greater export of nutrients in the most erosive rainfall events. However, it can be observed that after the low-intensity events, there were no significant differences between treated and non-treated soils. In this regard, Korboulewsky et al. (2002) indicated a potential increase in phosphorus release from compost-treated soils, resulting in concentrations that pose a risk to surface water. Morlat and Symoneaux (2008) indicated that mineralized N, following the application of high rates of organic amendments in vineyards, may exceed the needs of the vine, implying a risk of N leaching.

The results indicated that compost treatment could be beneficial because they increase infiltration and water storage in the soils, which means more water available for the crops. However, after high-intensity events, it may increase the risk of N and P export from the fields. Agriculture is considered one of the main sources of nonpoint pollution of P and N to surface waters (Carpenter et al., 1998; Wang et al., 2018), and vineyards are among the land uses that have a particular contribution to that diffuse source of pollution (Ferreira et al., 2018). After the high-intensity events, nutrient concentrations in runoff reached high values (above 20 mg L^−1^ for P and above 50 mg L^−1^ for N). According to phosphorus quality criteria (USEPA, 1986), phosphates should not exceed 0.1 mg L^−1^ in streams or flowing waters that directly discharge to lakes or reservoirs to control algal growth. In addition, for nitrogen, a total nitrogen concentration > 1.5 mg L^−1^ has been defined as a high level of eutrophication risk to water (Grizzetti et al., 2011). Although some of the N and P released and transported could be deposited in the field and not delivered to streams, the values observed greatly exceed the risk limits after most of the events, in particular after the high-intensity events. There is a big concern worldwide about N and P export by erosion and the effects of extreme storms on it (Ross et al., 2019; Shrestha et al., 2020) and its eventual disposition in the environment (Shi & Schulin, 2018; Sandström et al., 2020; Sishu et al., 2021). This research provides useful information on N and P export in vineyards under different management, which led to an enrichment in these elements at the soil surface. Furthermore, the results highlight the role of rainfall intensity on N and P export and that compost amendments could pose an increasing threat to the environment when high-intensity rainfall occurs in soils enriched with these elements.

## Conclusions

Compost amendment on rainfed vineyard soils improved hydrological properties of the soil. Steady infiltration rates increase and water holding capacity is also enhanced, which consequently leads to a decrease in runoff rates. However, the higher N and P contents at the soil surface lead to a higher nutrient release and higher concentration of nutrients in runoff. Despite the decrease in runoff rates, total N and P losses result higher in treated than in non-treated soils, in particular after events of high intensity which is the rainfall characteristic, responsible for rainfall erosivity, that mainly drives soil and nutrient losses and is the factor that marked significant differences between treated and non-treated soils. Therefore, to avoid an increase of N and P pollution in streams, the dose, timing, and/or frequency of amendment application should be controlled. The information, extracted after events of high intensity, may be of interest in view of the expected increase of high-intensity events due to climate change, in order to design strategies to preserve not only the soil and vineyards but also to protect the environment.

## Data Availability

Data supporting the results reported may be accessible by request.
